# CT utilization abruptly increases at age 18 among patients with inflammatory bowel diseases in the hospital

**DOI:** 10.1371/journal.pone.0195022

**Published:** 2018-03-29

**Authors:** Shail M. Govani, Peter D. R. Higgins, Joel H. Rubenstein, Ryan W. Stidham, Akbar K. Waljee

**Affiliations:** 1 Department of Internal Medicine, University of Michigan, Ann Arbor, MI, United States of America; 2 South Texas Veterans Healthcare System, San Antonio, Texas, United States of America; 3 UT Health San Antonio, San Antonio, Texas, United States of America; 4 VA Center for Clinical Management Research, VA Ann Arbor Health Care System, Ann Arbor, Michigan, United States of America; University Hospital Llandough, UNITED KINGDOM

## Abstract

**Objectives:**

Patients with inflammatory bowel disease(IBD) are frequently exposed to computed tomography (CT). Each CT exposes patients to radiation that cumulatively could increase the risk of malignancy, particularly in younger patients. We aim to study the effect of age on CT use in IBD patients seen in the Emergency Department (ED) or the hospital.

**Methods:**

We conducted a retrospective cohort study of IBD patients identified in Truven Health Marketscan databases between 2009–2013. The main outcome was use of CT during an ED or inpatient visit. Effect of age on CT use was characterized using logistic regression accounting for important covariables.

**Results:**

There were 66,731 patients with IBD with 144,147 ED or inpatient visits in this cohort with a diagnosis code of IBD. At first visit, 5.8% percent were below age 18. CT was utilized in 26.6% of visits. In multivariable analysis, adjusting for medications, recent surgery, and gender, patients 18–35 were more likely to undergo CT (OR 2.35, 95%CI: 2.20–2.52) compared to those <18. Examining patients only between 16 and 19, the odds of an 18 or 19-year-old undergoing CT is significantly higher than a 16 or 17-year-old (OR 1.96, 95%CI: 1.71–2.24).

**Conclusions:**

Patients with IBD undergo CT more than a quarter of the time in the ED or inpatient setting. Pediatric providers limit radiation exposure among those <18 while adult providers are not as cautious with radiation exposure for the young adult population. Increased awareness of the risks of cumulative radiation exposure in the young adult population is needed.

## Background

Inflammatory bowel disease (IBD) is a chronic relapsing and remitting autoimmune disorder of the gastrointestinal tract. IBD often presents at a young age, with a majority of patients diagnosed before age 30[1,2]. It is associated with significant morbidity with complications that include toxic megacolon, abscesses, fistulas, and obstructions. Computed tomography (CT) is an important tool in the diagnosis of such complications[3,4] and is increasingly used as this technology becomes more readily available[5]. However, the benefits of CT use must be balanced with the radiation exposure, which is particularly problematic as patients with IBD are frequently exposed to repeated CT. Based on data from atomic bomb survivors and nuclear industry workers, radiation exposures over 50mSv are associated with an increased risk of malignancy[6]. This risk is particularly great for patients who are exposed to radiation at young ages. Radiation exposures from CT of the abdomen and pelvis are estimated to be between 4-45mSv (median 16mSv)[7]. Patients with IBD are at risk for repeated radiation exposures from CT so as a result, cohort studies have found that up to 25% of patients with IBD have been exposed to more than 50mSv [8]. Radiation exposure in the hospital setting makes up a significant amount of this exposure. Seventy-five percent of radiation exposure in this patient population has been attributed to CT scans with 34% of CT scans performed in the emergency department (ED) and 31% of Crohn’s disease patients who are admitted undergoing CT[9]. These cumulative exposures are receiving increased attention as an important source of unnecessary radiation exposure[10]. Campaigns like “Image Gently” have improved recognition of this issue in the pediatric population through increasing education for providers and patient’s families[11,12]. While the importance of limiting radiation exposure is important among the pediatric population, the increased risk of malignancy due to prior radiation exposure extends far beyond age 18 to age 35[6] at which point, the risk of further radiation exposure is significantly lower.

We aimed to compare the prevalence of CT between adult and pediatric populations in the ED and inpatient setting using an administrative database. We hypothesized that pediatric populations were much less likely to undergo CT compared to the adults and that this imaging utilization occurred abruptly at age 18, rather than at age 35 when the radiation exposure risks are significantly lower.

## Methods

We conducted a study of the Truven Health MarketScan Commercial Claims and Encounters database from 2009 to 2013[13] looking for patients with a hospitalization or ED visit with a primary or secondary diagnosis of IBD based on ICD-9CM coding. This large administrative database is derived primarily from data from insurance coverage of patients who are covered by large employers in the United States. The database covers inpatient and outpatient visits in addition to pharmacy claims data of approximately 50 million active employees, early retirees, Medicare-eligible retirees with employer-provided Medicare supplemental plans, and their dependents each year. The data is anonymized. Informed consent is not obtained from the patients included in the database. The database can be obtained by contacting lifesciences@truvenhealth.com.

Patients were identified as carrying a diagnosis of IBD based on the presence of at least 1 inpatient visit or 2 outpatient visits with an IBD diagnosis (555 or 556) and at least 12 months of uninterrupted coverage. Patients without pharmacy benefits were excluded as we could not adjust for medication use without this data. The specific IBD diagnosis (Crohn’s disease or ulcerative colitis) was assigned if all of the patient’s visit coding was consistent with the appropriate ICD-9CM code. In instances which the diagnosis was not consistent, a diagnosis of indeterminate colitis was assigned[14]. We classified patients as having an ED visit if a current procedural terminology (CPT) code for emergency department services was billed (see list in S1 Appendix). Similarly, CT and surgery codes were classified based on CPT codes detailed in the supplementary appendix.

We integrated the pharmaceutical dispensing records with the inpatient and ED visit records to determine the use of medications at the time of ED or inpatient stays to control for severity of disease. We also included recent IBD surgery as a covariable as this has been identified as a predictor of significant findings on CT[15]. Anti-TNFs (infliximab, adalimumab, certolizumab or golimumab) and immunomodulators (thiopurines or methotrexate) use was characterized by a prescription overlap with the date of visit. Corticosteroid (prednisone) use and narcotic (see list in S2 Appendix) use was characterized by a prescription which would have supplied drug to within 90 days of the admission or ED visit. Only prescriptions supplying 20 days of narcotics or more were included in the analysis. Records which indicated a significant oversupply (>10%) of medications (ratio of days supplied to days covered) were deleted as these were suspected to represent errors in the dataset. For patients who were hospitalized, a Charlson-Deyo comorbidity index was calculated at each visit to adjust for other important comorbidities[16]. We chose this score because it is commonly used in the analysis of administrative databases to adjust for other concurrent illnesses. The University of Michigan Institutional Review Board approved this study (HUM00101283).

### Statistical analysis

Descriptive statistics were calculated using SAS 9.4 (Cary, NC). Comparisons were made between the patients who underwent CT and those who did not using either the Student’s t-test or chi square test. P values were obtained by comparing the appropriate statistic and the associated estimated variability based on the sample size (n). All statistical tests were 2-sided with a p value of <0.05 considered statistically significant. Logistic regression was used to determine the univariable and multivariable odds of CT during an ED or inpatient admission among the patients with a first or second diagnosis code of IBD and with prescription coverage controlling for other important predictors. To evaluate the effect of age on CT use, age was characterized as <18, 18–35 and >35 with the patients in the youngest age group considered the reference range. We selected these age ranges because 18 is typically the age limit for evaluation in a pediatric setting and 35 is the age limit at which further radiation exposure is believed to have little effect on malignancy risk. Multivariable models were constructed with all available variables to attempt to control for disease severity. To evaluate if a threshold existed at age 18 specifically, the predicted probability of CT use at each visit was calculated maintaining the other variables in a multivariable model constant to display the effect of age on CT use with age in this instance considered a continuous variable. A sensitivity analysis was performed to ensure that the effect of age did not change with the inclusion of multiple visits per individual. A random visit was chosen for those patients with multiple visits so that only one visit per individual was included in the multivariable model. Additionally, a sub-analysis was performed using multivariable logistic regression examining only patients between age 16 and 19 to determine the odds of CT use for those > = 18 compared to those <18 to further demonstrate the change in risk of CT at age 18.

## Results

There were 246,757 patients with a diagnosis of IBD and among these, 191,915 patients had pharmaceutical coverage in our cohort. Of these, 66,731 individual patients visited the emergency department (ED) or were hospitalized with a total of 144,147 ED visits or hospitalizations. Among the patients, 41.4% of the patients had Crohn’s versus 23.4% with ulcerative colitis. The remaining 35.2% were classified as indeterminate colitis. Among the population, 5.8% were under the age of 18 at the first IBD visit and 56.8% were female. In this cohort, 60.4% underwent no CT imaging in the study period, 29.8% underwent 1 CT during the study period and the remaining 9.2% underwent 2 or more CTs. The maximum number of CT exposures was 30. Examining these visits, the average number of CTs was significantly higher in the adult population compared to the pediatric population (1.5 versus 1.3, p<0.001). CT was utilized in 26.6% of ED visits or inpatient stays. A comparison of the individuals who underwent CT scans and those who did not found no difference in age, anti-TNF use, narcotic use and steroid use but did find that females and patients with UC were less likely to undergo CT (Table 1). Patients who did undergo CT had longer lengths of stay and were less likely to be on immunomodulators.

**Table 1:** Comparison of CT use among ED visits and hospitalizations for patients with IBD with pharmaceutical coverage.

	No CT (n = 105,782)	CT (n = 38,365)	Test Statistic(DF)	Test Value	p value
Age, mean (SD)	43.7 (18.5)	43.7 (16.1)	t(144145)	0.09	0.92
Female (%)	61,171 (57.8)	21,753 (56.7)	C2(1)	14.64	<0.001
Diagnosis Crohn's Diagnosis (%)	46,455 (43.9)	17,360 (45.3)	C2(2)	564.30	<0.001
Ulcerative Colitis (%)	20,852 (19.7)	5,525 (14.4)			
Indeterminate Colitis (%)	38,475 (36.4)	15,480 (40.4)			
Anti-TNF use at time of visit (%)	5,599 (5.3)	2,117 (5.5)	C2(1)	2.82	0.09
Narcotic use in previous 90 days (%)	37,779 (35.7)	13,612 (35.5)	C2(1)	0.67	0.41
Immunomodulator use (%)	8,180 (7.7)	2,833 (7.4)	C2(1)	4.85	0.03
Steroid use in prior 90 days (%)	12,143 (11.5)	4,428 (11.5)	C2(1)	0.10	0.74
Length of stay in days, mean (SD)	4.8 (6.1)	5.9 (6.9)	t(102968)	-25.16	<0.001

Using logistic regression, the odds ratio of a patient age 18–35 undergoing CT was 2.19 (95%CI 2.06–2.33) compared to those below age 18 (Table 2). Comparing patients older than 35 to those below 18, the odds ratio of CT was 2.11 (95%CI: 1.99–2.24). Females were slightly less likely to undergo CT (OR 0.96, 95%CI: 0.93–0.98). Patients with a recent surgery and those with a diagnosis of CD were more likely to undergo CT imaging (Table 2). There was no observed effect of time on CT use in univariable analysis (OR = 1.00, 95%CI: 0.99–1.01). A sub-analysis of the patients between 16 and 19 found that those who were ages 18–19 were significantly more likely to undergo CT than those who were ages 16–17 (OR 1.73, 95%CI: 1.55–1.95).

**Table 2:** Univariable predictors of CT use during an Inpatient or ED visit with a first or second diagnosis of IBD.

Predictor	OR	95%CI	p value
Age			
<18	1	Reference	
18–35 (versus <18)	2.19	2.06–2.33	<0.001
>35 (versus <18)	2.11	1.99–2.24	<0.001
Gender (F vs M)	0.96	0.93–0.98	<0.001
Current anti-TNF Use	1.05	0.99–1.10	0.09
Current Immunomodulator Use	0.95	0.91–1.00	0.03
Recent Narcotic Use (90 days)	0.99	0.97–1.01	0.41
Recent Surgery (Last 30 days)	1.87	1.67–2.09	<0.001
Steroid Use (Last 90 days)	1.01	0.97–1.04	0.74
Crohn's Diagnosis (vs. UC)	1.41	1.36–1.46	<0.001
Charlson-Deyo (per 1 point)	0.87	0.85–0.88	<0.001
Age 18–19 compared to 16–17	1.73	1.55–1.95	<0.001
Visit Year	1	0.99–1.01	0.75

**Fig 1:**
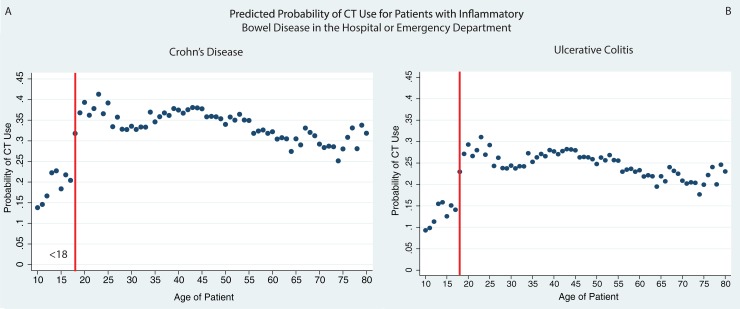
CT utilization by age among patients with IBD in the hospital or ED. Predicted probability of CT use by age demonstrates an abrupt increase at age 18 (vertical red line) for a male with Crohn’s disease (1A) or ulcerative colitis (1B) on no medications and with no recent surgery.

In multivariable analysis of CT use, the relationship between age and CT scan use persisted (Table 3). Controlling for sex, IBD diagnosis, medication use (current anti-TNFs and immunomodulators, recent narcotics and steroids), recent surgery and Charlson-Deyo comorbidity score, patients ages 18–35 much more likely to undergo CT during their inpatient or ED visit (OR 2.36, 95%CI: 2.20–2.53) compared to those <18. The predicted probabilities of CT use for each age among men with Crohn’s disease and UC and no comorbidities, no recent surgery, and on no steroids, immunosuppressives or anti-TNFs shows that the use of CT abruptly increases at age 18 (Fig 1). The sensitivity analysis including only 1 random visit for all individuals found that the odds of an 18–35 year old undergoing CT compared to a patient under the age of 18 remained high (OR: 2.07, 95%CI: 1.86–2.29, S3 Appendix). A sub-analysis of the patients age 16–19 found that the odds of CT use among patients 18–19 was 1.96 (95%CI: 1.71–2.24), compared to those 16–17 adjusting for the same covariates. The p values of the age association on CT use were all <0.001, which even accounting for all the test performed would be significant if accounting for a Bonferroni correction (0.05/21 = 0.002).

**Table 3:** Multivariable predictors of CT use during an inpatient or ED visit with a first or second diagnosis of IBD.

Predictor	OR	95%CI	p value
Age			
<18	1	Reference	
18–35 (versus <18)	2.35	2.20–2.52	<0.001
>35 (versus <18)	2.21	2.07–2.36	<0.001
Gender (F vs M)	0.94	0.92–0.97	<0.001
Current anti-TNF Use	1	0.94–1.06	0.89
Current Immunomodulator Use	0.95	0.90–1.00	0.04
Recent Narcotic Use (90 days)	0.94	0.91–0.97	<0.001
Recent Surgery (Last 30 days)	2.14	1.88–2.43	<0.001
Steroid Use (Last 90 days)	1.03	0.99–1.08	0.15
Crohn's Diagnosis (vs. UC)	1.59	1.53–1.66	<0.001
Charlson-Deyo (per 1 point)	0.87	0.85–0.88	<0.001

## Discussion

Patients with IBD frequently undergo cross sectional imaging as part of their inpatient and ED visits[5,15,17–19]. Prior studies of a tertiary care center in the United States [5] found that CT use increased in the ED setting from 25.8% to 40.2% of visits between 2000 and 2010 among patients with Crohn’s disease. In this administrative database, the rate of CT scan use among patients with a diagnosis of IBD was 26%. Patients under the age of 18 were far less likely to undergo CT scan than those above the age of 18 despite controlling for disease type, gender, recent surgery and medications.

Cross sectional imaging plays an important role in the management of IBD[4], but the use of CT can be associated with significant radiation exposure. A study of radiation exposure per CT of the abdomen/pelvis with contrast found an average exposure of 16mSv[7]. Another study of CT use from 5 centers in the US in 2013 found similar exposures but did note that children were typically exposed to 75% less radiation.[20] While there have been significant efforts to reduce individual CT radiation doses[21], patients with IBD tend to be exposed to CT repeatedly, leading to large cumulative radiation exposures. Approximately 8% of patients with IBD were exposed to more than 50mSv[22] in a meta-analysis performed in 2012. Patient with Crohn’s disease are typically exposed to more radiation[9] with 10% of patients with Crohn’s disease being exposed to more than 75mSv of radiation and that risk factors associated with a higher cumulative exposure includes prior surgeries, anti-TNF use, early diagnosis (before age 17), and need for IV steroids[23]. In Olmsted county, the median radiation exposure was 26mSv for Crohn’s patients, but the exposure for the top quartile ranged from 48 to 279 mSv over a 21 year period suggesting that a subset of IBD patients are exposed to very large amounts of radiation[8]. During the 5 years that our study covered, we found similar findings with 9% of the patient population undergoing 2 CTs or more and 60% of the patients undergoing no CT scans.

Pediatric radiologists have followed the principle of “As Low As Reasonably Achievable” (ALARA) in the development of imaging protocols. The issue has been increasingly important as CT use increased by 800% since the 1980s among children[12]. The “Image Gently” campaign was launched in 2008 to increase awareness of radiation exposure among radiologists, technicians and other providers. Despite this campaign, survey studies have found that awareness of radiation exposure among providers and patients remains very low[24,25]. A recent survey of ED providers identified that a majority thought CT was overused, but most significantly underestimated the radiation exposure per CT[26]. Interestingly, more than 80% of the providers surveyed felt that age 20 and below was the threshold at which they would consider another study. Our results appear to confirm this threshold effect on radiation risks and subsequent CT use. While the risk of cancer attributed to radiation does decrease with age, the slope of decreasing risk does not flatten out until age 35[27], suggesting that ordering providers should be more cognizant of repeated CTs for patients between 18 and 35.

We also note that providers are less likely to use CT for females than males in this cohort. It is possible that providers were more concerned about radiation exposure among females or that there were other concerns including pregnancy which prevented the use CT among women. A prior meta-analysis did not identify a relationship between gender and high utilization of CT [22]. Our study is from a later time period than the studies in the meta-analysis and may reflect changing attitudes of radiation risks on females versus males.

Providers have 2 options which do not involve radiation to assess for complications related to IBD: MRI and ultrasound. Both of these modalities have similar diagnostic characteristics to detect complications [28]. MRI is more expensive than CT, requires contrast ingestion, has a longer acquisition time and requires specialized expertise for interpretation and therefore is not as readily available in the ED. If imaging can be deferred until the patient is hospitalized, MRI would be the preferred alternative. Ultrasound on the other hand can be done in real-time and is cheaper than both CT and MRI. The caveats of ultrasound are that there is significant inter-operator variability, small field of view and limited views of the deep abdomen, especially in an obese patient [29]. While ultrasound is becoming a more common tool in the evaluation of IBD patients in Europe, it is not commonly used in the United States due to lack of training for this indication. With time, MRI and ultrasound will hopefully become more widely available and utilized for IBD.

The major strengths of our study include the comprehensive nature of the insurance dataset contained in Marketscan. Studies of an individual institution’s CT use may miss CTs obtained at other institutions. This analysis is also more generalizable to community practice since it is not limited to tertiary care centers. Our study is limited in that patients under the age of 18 may be more likely to have state-funded insurance and therefore not be captured in this dataset. This administrative dataset also lacks granular data, specifically data regarding the CT results or prior CT results that may factor in the decision to pursue more imaging. There were repeated measures of 10% of the individuals in our dataset present. We attempted to control for this by selecting only 1 random visit per individual and found that patients over the age of 18 were still more than twice as likely to undergo CT as those below the age of 18. We also do not have data on the amount of radiation exposure per CT obtained. It is possible that patients between 18 and 35 were subjected to low dose radiation protocols which may reduce the risk of radiation exposure. We attempted to control for disease severity using medication use but we were unable to control for disease duration or prior history of complications reliably which may factor into the decision to use CT. Other factors such as the specialty of the ordering provider or type of hospital also could not be adjusted for in our model. Pediatric trained ED providers or visits in a pediatric ED may factor into CT use. While the median CT exposure was only 1, our study only covered a 5-year period, suggesting that exposures over longer periods of time maybe more meaningful.

In conclusion, CT use among patients with IBD in the ED or hospitalized increases significantly at age 18. Despite the fact that significant radiation exposure risks persist up to age 35, there is a major and abrupt increase in CT use at age 18. Further work is necessary to increase awareness of the risk of radiation exposure among young adults and to increase the use of alternatives to CT such as ultrasound or MRI in this population.

## Supporting information

S1 Appendix CPT Codes for ED visits, surgery and CT scans. (DOCX)

S2 Appendix Narcotic medication list. (DOCX)

S3 Appendix Supplementary Table 1. Multivariable model of CT Use with 1 random visit per individual.
(DOCX)
