# Supplementary material for: CT utilization abruptly increases at age 18 among patients with inflammatory bowel diseases in the hospital
Source: PLoS One. 2018 Mar 29;13(3):e0195022. doi: 10.1371/journal.pone.0195022 (PMC5875842; doi:10.1371/journal.pone.0195022)
Supplement: S1 Appendix — (DOCX) [file pone.0195022.s001.docx]

CPT codes for Emergency Department Visits: 99285, 99284, 99283, 99282, 99281

CPT codes for surgery: 49000, 44005, 44120, 44125,44140, 44143, 44310, 44320, 49020, 49321, 46707, 46710, 46712, 46288, 46270, 46280, 46275, 45562, 45563, 44145, 44146, 44120, 44121, 45110, 45119, 45111, 45112, 45113, 45114, 45115, 45116, 45117, 45118, 44202, 44204, 44205, 44206, 44207, 44208, 44210, 44211, 44212, 44213, 44227, 45395, 45397, 45400, 45402

CPT codes for CT: 74177, 74178, 74176, 74160, 74150, 74170, 74174
