# Supplementary material for: CT utilization abruptly increases at age 18 among patients with inflammatory bowel diseases in the hospital
Source: PLoS One. 2018 Mar 29;13(3):e0195022. doi: 10.1371/journal.pone.0195022 (PMC5875842; doi:10.1371/journal.pone.0195022)
Supplement: S2 Appendix — (DOCX) [file pone.0195022.s002.docx]

Narcotic Medication List:

'Acetaminophen/Hydrocodone Bitartrate',

'Chlorpheniramine Polistirex/Hydrocodone Polistirex',

'Guaifenesin/Hydrocodone Bitartrate',

'Homatropine Methylbromide/Hydrocodone Bitartrate',

'Hydrocodone Bitartrate',

'Hydrocodone Bitartrate/Ibuprofen',

'ASA/Oxycodone HCl/Oxycodone Terephthalate',

'Acetaminophen/Oxycodone Hydrochloride',

'Aspirin/Oxycodone Hydrochloride',

'Ibuprofen/Oxycodone Hydrochloride',

'Oxycodone Hydrochloride',

'Acetaminophen/Tramadol Hydrochloride',

'Tramadol Hydrochloride',

'Propoxyphene Hydrochloride',

'Propoxyphene Napsylate',

'Codeine Phos/Dexchlorpheniramine Mal/Phenyleph HCl',

'Codeine Phos/GG/PSE HCl',

'Codeine Phos/Phenyleph HCl/Promethazine HCl',

'Codeine Phos/Phenyleph HCl/Pyril Mal',

'Codeine Phosphate/Guaifenesin',

'Codeine Phosphate/Phenylephrine Hydrochloride',

'Codeine Phosphate/Promethazine Hydrochloride',

'Codeine Phosphate/Pseudoephedrine Hydrochloride',

'Chlorpheniramine Maleate/Codeine Phosphate',

'CPM/Dihydrocodeine Bitartrate/PSE HCl',

'CPM/Dihydrocodeine Bitartrate/Phenyleph HCl',

'CPM/Codeine Phos/PSE HCl',

'BPM/Codeine Phos/PSE HCl',

'BPM/Codeine Phos/Phenyleph HCl',

'Aspirin/Codeine Phosphate',

'Aspirin/Carisoprodol/Codeine Phosphate',

'Aspirin/Butalbital/Caffeine/Codeine Phosphate',

'Acetaminophen/Codeine Phosphate',

'APAP/Butalbital/Caff/Codeine Phos',

'Hydromorphone Hydrochloride',

'Meperidine HCl/Promethazine HCl',

'Meperidine Hydrochloride',

'Morphine Sulfate',

'Morphine Sulfate/Naltrexone Hydrochloride',

'Fentanyl',

'Fentanyl Citrate',

'Acetaminophen/Caffeine/Dihydrocodeine Bitartrate',

'BPM/Dihydrocodeine Bitartrate/PSE HCl',

'BPM/Dihydrocodeine Bitartrate/Phenyleph HCl',

'CPM/Dihydrocodeine Bitartrate/PSE HCl',

'CPM/Dihydrocodeine Bitartrate/Phenyleph HCl',

'Dihydrocodeine Bitartrate/GG/PSE HCl',

'Dihydrocodeine Bitartrate/GG/Phenyleph HCl',

'Dihydrocodeine Bitartrate/Guaifenesin',

'Dihydrocodeine Bitartrate/Phenyleph HCl',

'Acetaminophen/Pentazocine Hydrochloride',

'Naloxone Hydrochloride/Pentazocine Hydrochloride',

'Oxymorphone Hydrochloride',

'Tapentadol Hydrochloride’
