# Supplementary material for: CT utilization abruptly increases at age 18 among patients with inflammatory bowel diseases in the hospital
Source: PLoS One. 2018 Mar 29;13(3):e0195022. doi: 10.1371/journal.pone.0195022 (PMC5875842; doi:10.1371/journal.pone.0195022)
Supplement: S3 Appendix — Multivariable model of CT Use with 1 random visit per individual. (DOCX) [file pone.0195022.s003.docx]

**Supplementary Table 1.** Multivariable Predictors of CT Use During an Inpatient or ED Visit with a First or Second Diagnosis of IBD with Only 1 Random Visit per Individual

| Predictor |  | OR | 95%CI | p value |
| --- | --- | --- | --- | --- |
| Age |  |  |  |  |
|  | <18 | 1 | Reference |  |
|  | 18-35 | 2.07 | 1.86-2.29 | <0.001 |
|  | >35 | 2.01 | 1.82-2.22 | <0.001 |
| Gender (F vs M)  Current anti-TNF Use  Current Immunomodulator Use  Recent Narcotic Use (90 days)  Recent Surgery (Last 30 days)  Steroid Use (Last 90 days)  Crohn's Diagnosis (vs. UC)  Charlson-Deyo (per 1 point) | |  |  |  |
|  |  | 0.92 | 0.88-0.95 | <0.001 |
|  |  | 0.87 | 0.79-0.97 | 0.01 |
|  |  | 0.88 | 0.81-0.95 | 0.001 |
|  |  | 0.90 | 0.86-0.94 | <0.001 |
|  |  | 2.34 | 1.81-3.03 | <0.001 |
|  |  | 1.04 | 0.97-1.10 | 0.31 |
|  |  | 1.56 | 1.48-1.65 | <0.001 |
|  |  | 0.88 | 0.86-0.90 | <0.001 |
